# Meniscal Extrusion in the Knee: Should only 3 mm Extrusion be Considered Significant? An Assessment by MRI and Arthroscopy

**DOI:** 10.5704/MOJ.1507.013

**Published:** 2015-07

**Authors:** N Muzaffar, O Kirmani, M Ahsan, S Ahmad

**Affiliations:** Hospital for Bone and Joint Surgery, Srinagar, India; * Department of Radiodiagnosis, Government Medical College, Srinagar, India

**Keywords:** Extrusion, Meniscal tear, MR imaging, Knee

## Abstract

Aim. The aim of this study was to assess whether significant meniscal extrusion of more than 3 mm or of even lesser degrees of extrusion could be considered significant. We also aimed to determine the morphology of tears that are most likely to be associated with significant extrusion.

Study design and material. The study was done retrospectively on a group of 202 patients (157 males and 45 females) who had been seen in our hospital between 2007 and 2011 with meniscal tears (in one knee only) diagnosed by MRI and confirmed on arthroscopy. Extrusion of 3 mm or more (usually considered significant) was seen in 102 cases and less than 3 mm in 100. Extrusion was measured on the coronal MR images rather than on saggital images because of ease and reproducibility. The tears were confirmed by arthroscopy and correlated with the extent of extrusion on MRI.

Results: Out of the total of 202 cases, 102 cases (50.5%) had extrusion of 3 mm or more on MRI. Of these, the medial meniscal posterior horn tears accounted for 63 cases (64.26%), 21 cases were medial meniscal body tears (21.42%), five medial meniscal root tears (5.1%), nine lateral meniscal body tears (9.18%) and four lateral meniscal posterior horn tears(4.08%). Forty-four cases had extrusion of 3-4 mm, 26 had extrusion of 4-5mm, 17 cases had extrusion of 5-6mm, ten had extrusion of 6-7mm and five had extrusion of 7 mm or more. One hundred cases fell in the < 3mm extrusion category, of which 80 (39.6%) were in the 2-3 mm extrusion group and 20 (9.9%) in the 1-2 mm extrusion group. They comprised of 61 cases of medial meniscal posterior horn tears, 23 cases of medial meniscal body tears, six medial meniscal root tears, eight lateral meniscal body tears and two lateral meniscal posterior horn tears. The highest proportion of meniscal tears was seen in the 2-3 mm category comprising nearly 40% of the entire study group. The majority of tears were medial meniscal posterior horn tears.

Conclusion: Menisci that extruded 2-3 mm from the tibial margin formed a major proportion of menisci treated for tears by repair or menisectomy. We should consider extrusion of more than 2mm as significant. Most tears had extrusion of 2-4 mm.

## Introduction

Meniscal extrusion occurs due to substantial disruption of the main circumferential collagen bundle fibers in the meniscus. Tears resulting in extrusion include meniscal horn/root tears, radial tears of more than 50% of meniscal width in size and large complex tears (more than one cleavage plane through the meniscus)^1,2^. These result in loss of the ability to resist hoop strain and biomechanically overload the joint articular surface. Significant meniscal root pathology may cause functional incompetence of the meniscus, with consequent early onset cartilage degeneration and osteoarthritis. This study emphasizes the importance of the association of meniscal tear with the amount of extrusion of the meniscus beyond the tibial margin that can be considered statistically significant. We have also attempted to study the significant correlation of the meniscal tear morphology with its extrusion and to verify the MRI findings with arthroscopic examination. Our hypothesis is that extrusion beyond 2 mm of the joint margin should be considered significant, more so in medial meniscal posterior horn tears.

**Fig. 1a-b:**
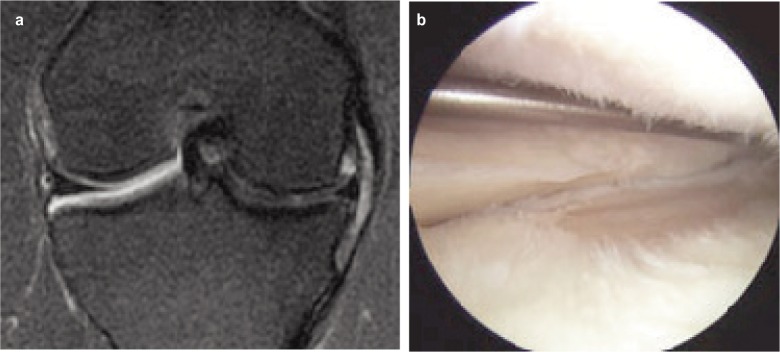
Meniscal extrusion on MRI and the arthroscopic image of the tear.

**Fig. 2a-c:**
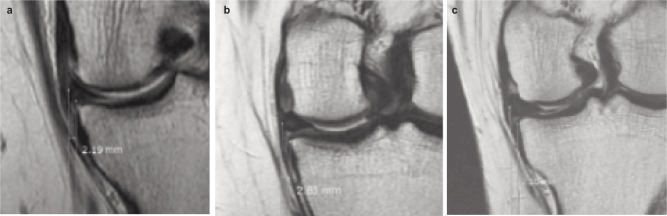
Meniscal extrusion less than 3 mm in three cases.

**Fig. 3:**
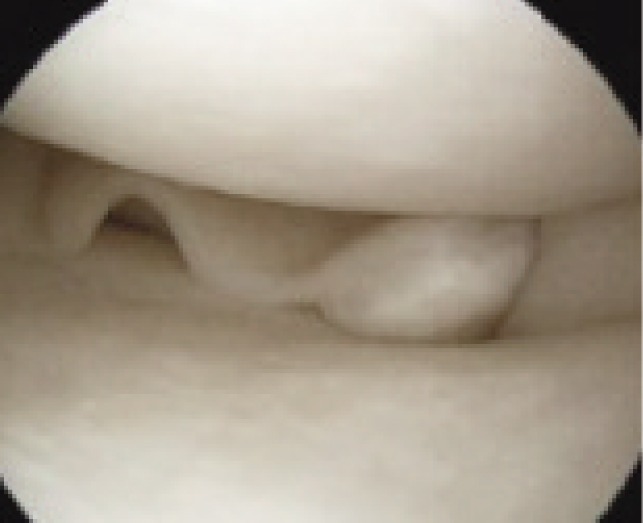
Meniscal tear on arthroscopy in case shown in Fig 2(a).

## Materials and Methods

This retrospective study was carried out between 2008 and 2012 on 202 patients who underwent MRI and subsequent arthroscopy at our hospital for meniscal tears and associated pathology. There were 157 males and 45 females, with age range of 40-81 years (average 58 years). Only one knee was studied per patient. MRI examinations were performed with a 1.5-T scanner using a quadrature extremity coil. MR imaging incorporated the following sequences: sagittal spin-echo intermediate-weighted, sagittal and axial fast spin-echo T2-weighted with fat saturation and coronal fast spin-echo intermediate-weighted. For measurement of extrusion, only the coronal image at the mid point of the medial femoral condyle was assessed and extrusion of the meniscus from the margin of the tibial plateau was measured in millimeters using a PACS (Picture Archiving and Communication System) workstation. The measurement was performed by first drawing a vertical line intersecting the peripheral margin of the tibial plateau at the point of transition from horizontal to vertical; the length of another line extending from the first line to the outer margin of the meniscus was defined as the measurement of meniscal extrusion. All cases underwent arthroscopy performed by a single experienced arthroscopist within 4-6 weeks of the MRI.

## Results

The cohort of 202 cases included 157 males and 45 females in the age group of 40-81 years with mean = 58.361 years, 95% confidence interval for mean: 57.46 through 59.27 and standard deviation = 9.21, median = 58.00 and average absolute deviation from median = 7.00. Of these 202, there were 102 cases with extrusion of 3 mm or more (50.5%) on coronal MRI. Here, mean = 4.5965 mm with 95% confidence interval for mean: 3.322 through 5.871, standard deviation = 1.23 with a high = 8.280, low = 3.000, median = 4.220 and average absolute deviation from median = 0.965. Of these, the medial meniscal posterior horn tears accounted for 63 cases (64.26%), 21 cases were medial meniscal body tears (21.42%), five were medial meniscal root tears (5.1%), nine lateral meniscal body tears (9.18%) and four lateral meniscal posterior horn tears (4.08%).

Forty-four cases had extrusion of 3-4 mm, 26 had extrusion of 4-5mm, 17 cases had extrusion of 5-6mm, 10 cases had extrusion of 6-7mm and 5 cases had extrusion of 7 mm or more. The remaining 100 cases fell in the < 3mm extrusion category and in this group, mean was 2.4104 mm with 95% confidence interval for mean: 1.123 through 3.698, standard deviation was 0.396 with high of 2.980 and low of 1.500.

Median=2.500 and average absolute deviation from median = 0.315. Out of these 100 cases, 61 were medial meniscal posterior horn tears, 23 cases of medial meniscal body tears, six medial meniscal root tears, eight lateral meniscal body tears and two lateral meniscal posterior horn tears. Eighty cases had 2-3 mm extrusion and 20 had 1-2 mm extrusion. In the 2-3 mm extrusion group, 47 cases were medial meniscal posterior horn tears, 18 were medial meniscal body tears, 13 - lateral meniscal tears and two medial meniscal root tears.

## Discussion

Meniscal extrusion is generally defined as significant (≥3 mm) medial displacement of the meniscus with respect to the central margin of the tibial plateau^2^. Detection of meniscal extrusion is important not only because it is associated with underlying tear but also because meniscal extrusion itself is thought to be related to development of osteoarthritis^3,4^. Medial meniscal extrusion is a significant finding on MRI, showing the inability of the meniscus to protect the underlying articular cartilage. In many studies, it has been shown to precede cartilage loss and onset of bony degenerative joint disease within the knee. Meniscal extrusion is the result of any substantial disruption of the main circumferential collagen bundles. Tears resulting in extrusion include meniscal root tear, large radial tear (more than 50% of meniscal width) and large complex tears (more than one cleavage plane through the meniscus)^1,2^. These result in loss of ability to resist hoop strain (circumferential stress). During load transmission, the compression forces working on the meniscus result in hoop strain that stretches the collagen bundles in a radial direction between the anterior and posterior attachments. The integrity and orientation of the meniscal collagen fibers, the attachments of anterior and posterior horns, and the presence of intermeniscal connections are some factors that influence resistance to hoop strain^5^.

Miller^6^ showed that extrusion greater than 25% of meniscal width was not significantly associated with meniscal tear. But he did not account for the high incidence of meniscal degeneration, which can disrupt meniscal mechanics. According to Kenny^4^, post meniscectomy, as a result of loss of meniscal function, the following are signs (Fairbank’s signs) apparent on standard AP and lateral radiographs of the knee: an antero-posterior osseous ridge projecting downward from the femoral condyle, generalized flattening of the marginal half of the femoral articular surface, and narrowing of the joint space which were hallmarks of knees with radial displacement (i.e., extrusion) of the medial meniscus and loss of meniscal function. Complete or subtotal meniscectomy also induces rapidly progressive osteoarthritis.

Magee^7^ showed a high prevalence of meniscal root tears in patients with meniscal extrusion on MR exam. Meniscal root tears are uncommon in patients without meniscal extrusion on MR exam. There may be a subset of patients in which the meniscal root is stretched rather than torn. Medial meniscal extrusion in patients more than 50 years of age may be associated with a meniscal “stretch” injury due to degeneration of the meniscus without a meniscal tear detectable on arthroscopy. These menisci may have increased laxity due to compromised meniscal collagen fibers. This may predispose the patient to premature osteoarthritis. Meniscal extrusion is caused by osteoarthritis in the elderly^8^ and by trauma in young individuals^9,10^. Costa^1^reported that the degree of extrusion was significantly related to meniscal degeneration and that the most common extrusion was in the medial meniscus. The reason for extrusion being more common in the medial meniscus may be related to the anatomical structure and the medial meniscus being the point of weight-bearing. Tears involving the meniscal root (central attachment) are also significantly related to the severity of meniscal extrusion, seen in 3% with minor extrusion and 42% with major extrusion.

With meniscus extrusion, the meniscus is unable to resist hoop stresses and cannot shield the adjacent articular cartilage from excessive axial load.

Over time, this can lead to symptomatic knee osteoarthritis. Tears of the posterior meniscal root can be easily missed because of inconsistent clinical symptoms and can be overlooked without thorough arthroscopic examination.

Detection of meniscal extrusion is important not only because it is associated with underlying tear but also because meniscal extrusion itself is thought to be related to development of osteoarthritis.

**Conflict of interest:** The authors certify that they have no commercial associations (e.g., consultancies, stock ownership, equity interest, patent/licensing arrangements, etc.) that might pose a conflict of interest in connection with the submitted article. No financial aid was received for this study.

**Ethical Board Review statement:** This is to certify that the subjects gave informed consent to participate in the study and that the study has been approved by an institutional review board. The authors certify that this institute has approved or waived approval for the human protocol for this investigation and that all investigations were conducted in conformity with ethical principles of research.
